# Validation of the Model of End-Stage Liver Disease for Liver Transplant Allocation in Alberta: Implications for Future Directions in Canada

**DOI:** 10.1155/2016/1329532

**Published:** 2016-04-03

**Authors:** Kelly W. Burak, Glenda A. Meeberg, Robert P. Myers, Gordon H. Fick, Mark G. Swain, Vincent G. Bain, Norman M. Kneteman, Robert J. Hilsden

**Affiliations:** ^1^Liver Unit, Division of Gastroenterology and Hepatology, Department of Medicine, University of Calgary, Calgary, AB, Canada T2N 4Z6; ^2^Alberta Liver Transplant Program, University of Alberta, Edmonton, AB, Canada; ^3^Department of Community Health Sciences, University of Calgary, Calgary, AB, Canada

## Abstract

*Background*. Since 2002, the Model of End-Stage Liver Disease (MELD) has been used for allocation of liver transplants (LT) in the USA. In Canada, livers were allocated by the CanWAIT algorithm. The aim of this study was to compare the abilities of MELD, Child-Pugh (CP), and CanWAIT status to predict 3-month and 1-year mortality before LT in Canadian patients and to describe the use of MELD in Canada.* Methods*. Validation of MELD was performed in 320 patients listed for LT in Alberta (1998–2002). In October 2014, a survey of MELD use by Canadian LT centers was conducted.* Results*. Within 1 year of listing, 47 patients were removed from the waiting list (29 deaths, 18 too ill for LT). Using logistic regression, the MELD and CP were better than the CanWAIT at predicting 3-month (AUROC: 0.79, 0.78, and 0.59; *p* = 0.0002) and 1-year waitlist mortality (AUROC: 0.70, 0.70, and 0.55; *p* = 0.0023). Beginning in 2004, MELD began to be adopted by Canadian LT programs but its use was not standardized.* Conclusions*. Compared with the CanWAIT system, the MELD score was significantly better at predicting LT waitlist mortality. MELD-sodium (MELD-Na) has now been adopted for LT allocation in Canada.

## 1. Introduction

**Figure 1:**
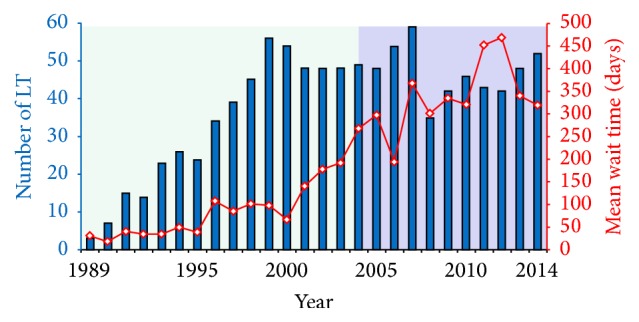
Number of adult cadaveric liver transplants performed per year (bars) and the mean waiting time in days (lines) for liver transplant in Alberta (1989–2014).

Liver transplant (LT) is often the only life-extending option for patients with acute liver failure (ALF) and complications of chronic liver disease. In recent years, the demand for LT has dramatically increased, largely due to the burden of chronic hepatitis C virus (HCV) in Canada [1]. However, wait times for LT have significantly lengthened because the supply of cadaveric and live donor organs has not increased enough to meet this demand. In Alberta, we saw a fourfold increase in mean waiting time for adult cadaveric LT between 2000 and 2004 alone (Figure 1).

In 1998, the United Network of Organ Sharing (UNOS) adopted minimal listing criteria in the United States for patients to be placed on the LT waiting list [2]. Child-Pugh (CP) classification (Table 1) was used to place patients with chronic liver disease into three categories of disease severity. With many patients in each category, ties were then broken by length of time on the waiting list. In 2000, the US Department of Health and Human Services mandated the emphasis on waiting time be removed from the process of organ allocation [3]. UNOS adopted the Model of End-Stage Liver Disease (MELD) score in February 2002 as an objective means of allocating organs to the patients with the greatest need [3]. The MELD score (based on bilirubin, creatinine, and INR) was first used to predict survival after transjugular portosystemic intrahepatic shunting [4] and was later validated as a predictor of mortality in patients awaiting LT in the USA and Europe [5–8]. The MELD policy has resulted in fewer patients being listed for LT and fewer deaths on the waitlist without changing mortality rates following LT in the USA [9, 10]. The organ allocation system in Canada is based on the CanWAIT algorithm (Table 2), which ranks patients according to location (intensive care unit, hospital ward, or home), and similar to the previous CP based system in the USA, it relies heavily upon waiting time to break ties within categories. Therefore, the primary purpose of this research was to validate the ability of the MELD score to predict mortality in a cohort of Canadian patients and to determine if MELD was superior to the CP score and CanWAIT status in predicting waitlist mortality.

In October 2005, the first annual Canadian Liver Transplant Forum (CLTF) was held in Montreal to address the question:* Should Canada adopt MELD for LT allocation*? Starting with Alberta in July 2004, LT programs in Canada began to adopt MELD for LT allocation locally for nonurgent status patients. However, MELD use in Canada has not been standardized and therefore the CLTF-9 meeting held in Montreal in October 2014 was once again focused on advancing consensus around listing criteria for LT in Canada.

## 2. Methods

**Figure 2:**
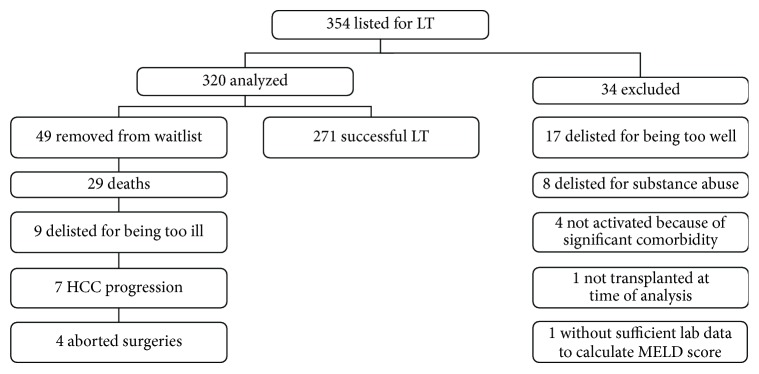
Flow chart of study subjects.

We examined a historical cohort of patients at the University of Alberta, from whom data had been collected prospectively into a database. Adult patients (>18 years old) who were listed for cadaveric LT between January 1, 1998, and December 31, 2002, were included in the study. Exclusion criteria included pediatric patients (<18 years old), previous solid organ transplants (including liver), simultaneous small bowel or renal transplants, live donor liver transplants (LDLT), patients who voluntarily removed themselves from the list, patients who recovered liver function (too well to need LT), patients who were delisted for active substance abuse or medical issues discovered prior to activation, and patients without complete laboratory data to calculate the MELD within 3 months of the listing date for LT (Figure 2). This study did include patients who were listed with fulminant acute liver failure (ALF = status 3F or 4F), although these patients receive preferential status and in the USA are not ranked by the MELD score. Also some of the hepatocellular carcinoma (HCC) patients were given preferential status (status 1T) beginning in 2001. Therefore, separate analysis was performed both including and excluding ALF and HCC patients.

The MELD score was calculated as per the original model [4] without the disease category:

(1) 0.957ln⁡creatinine+0.378ln⁡bilirubin+1.12ln⁡INR+0.643×10.

Creatinine and bilirubin were converted from *µ*mol/L to mg/dL (conversion factors 17.1 for bilirubin and 88.4 for creatinine). The MELD score was calculated as per UNOS guidelines, with the following exceptions: no extra points were awarded for HCC, and the score was not capped at 40.

The primary outcome variable in this study was mortality defined as death or delisting for being too ill. Logistic regression was used to examine the ability of MELD, CP, and CanWAIT status to predict 3-month and 1-year mortality on the LT waitlist. The models were compared using a chi-squared test for the equality of the area under the receiver operating characteristic (AUROC) curves [11]. The AUROC curve has become the most frequently performed statistical analysis in the validation studies of the MELD score [5–8]. The AUROC curve ranges from 0 to 1, with 1 representing perfect discrimination and 0.5 being due to chance alone. A diagnostic or prognostic test is generally accepted as clinically useful when the AUROC is ≥0.7 and AUROC of 0.8–0.9 indicates an excellent ability to predict an outcome. The ability of the MELD score to predict one-year waitlist survival was also examined using standard survival analysis techniques. Kaplan-Meier curves were compared using the log rank test. Hazard ratios were calculated using Cox proportional hazards models and formal testing was done to confirm that the assumption of proportional hazards was not violated. All statistical tests were performed using STATA 8.0 software. Tests of significance were two-sided with an alpha value of 0.05. Prior to initiating this project the Research Ethics Boards (REB) at the University of Calgary and the University of Alberta reviewed and approved this protocol.

In October 2014, a survey was conducted of all adult LT programs in Canada asking five questions:

1. When did you officially start allocating organs according to the MELD?
2. Do you still respect the CanWAIT status over MELD (does a hospitalized patient with a lower MELD score get an organ before a patient at home with a higher MELD score)?
3. What version of MELD do you currently use (MELD/MELD-Na/other)?
4. Describe how do you give MELD exemption points for HCC?
5. What other diagnoses regularly receive MELD exemption points in your centre?

These results were tabulated and presented at the CLTF-9 meeting in Montreal on October 18, 2014.

## 3. Results

### 3.1. MELD Validation

A total of 354 adults met inclusion criteria and were listed for LT in Alberta during the study period. Thirty-four subjects were excluded, leaving 320 patients for analysis (Figure 2). The mean age (±SD) of subjects listed was 50.2 ± 10.0 years and more males were listed than females (67.5% versus 32.5%). The most common indications for LT were alcoholic liver disease (33.4%) and HCV (32.8%) and only 15 subjects (4.7%) had ALF.

Table 3 shows the CanWAIT status at the time of listing. Nearly one-third of patients were listed as status 0 (pending further investigations before being activated). The status 0 group included patients who were later transplanted as status 1 (*n* = 57), status 1T (*n* = 4), status 2 (*n* = 22), status 4 (*n* = 6), and status 4F (*n* = 1). The median MELD score was 14 (range 5 to 49). Of the 320 subjects, 271 patients (84.7%) successfully underwent LT. A total of 49 patients (15.3%) were removed from the waitlist because of death or delisting for being too ill (Figure 2). Thirty-one removals occurred within 3 months of listing and 47 of the 49 removals occurred within one year of listing. The mortality rates on the waitlist by MELD strata, CP class, and CanWAIT categories are shown in Table 3. The mortality rates ranged from 7.6% for MELD scores ≤9 to 46.7% for MELD scores between 30 and 39. Within the CP classes, the highest mortality rates (25.2%) were seen in CP class C. Although the numbers were very small, patients with chronic liver disease on a ventilator (status 4) had the highest mortality rate within the CanWAIT system.

**Figure 3:**
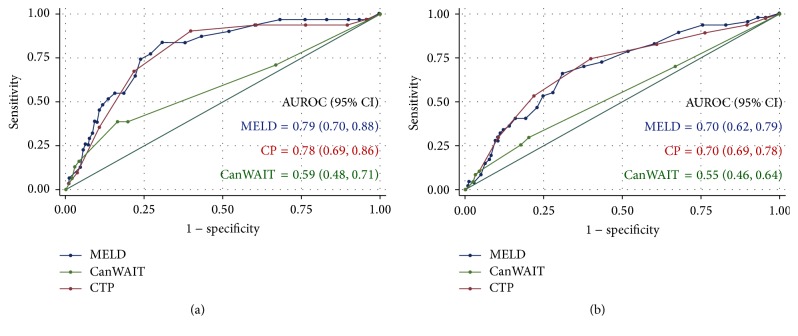
Receiver operating characteristic (ROC) curves for MELD, CP, and CanWAIT scores for prediction of 3-month (a) and 1-year (b) waiting list mortality.

The median MELD score in subjects who died within the first 3 months of listing was 21 (range 10 to 45) compared to a median MELD score of 15 (range 6 to 44) for those surviving (Mann-Whitney test, *p* = 0.018). The median MELD score in subjects who died within the first year of listing was 19 (range 6 to 43) compared to a median MELD score of 14 (range 5 to 49) for those surviving (*p* < 0.0005). The ROC curves for 3-month and 1-year waiting list mortality are shown in Figure 3. For the prediction of 3-month mortality the AUROC curves were similar for the MELD and CP scores (*p* = 0.70). The AUROC for the CanWAIT status was only 0.59 and was significantly lower than the AUROC curve for both the MELD (*p* = 0.0002) and CP (*p* = 0.0015) scores. For the prediction of 1-year waiting list mortality the AUROC curve was similar for the MELD and CP scores (*p* = 0.93). For the CanWAIT status it was only 0.55 and was significantly lower than the AUROC curve for both MELD (*p* = 0.0023) and CP (*p* = 0.006) scores. The ROC curves after excluding ALF and status 1T patients (*n* = 292) were nearly identical to the previous analysis (data not shown).

**Figure 4:**
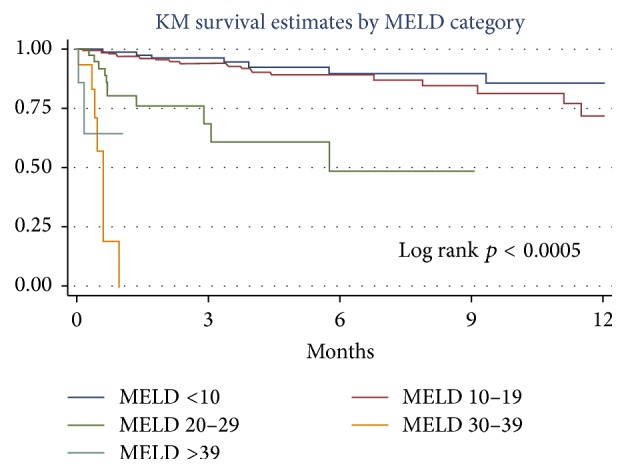
Kaplan-Meier survival estimates of 1-year waiting list survival for different strata of MELD scores.

The survival curves for the different MELD strata are shown in Figure 4. The log rank test for equality of survivor functions between different MELD categories was significant (*p* < 0.0005). Using the MELD <10 strata as the comparison group, only MELD strata ≥ 20 had significant hazard ratios for mortality at 1 year (Table 4).

### 3.2. MELD Use in Canada

LT allocation policy in Canada primarily focuses on sharing of organs for urgent status patients, which until May 2010 included statuses 3F, 4, and 4F, after which interprovincial sharing was restricted to only acute liver failure patients (statuses 3F and 4F). Center-specific variation for allocation of organs to nonurgent patients therefore exists. MELD began to be adopted in other jurisdictions in Canada starting in 2004 (Table 5); however, only British Columbia would take into account the patient's status (hospitalized versus home) when deciding who is next to receive a nonurgent transplant. As seen in Table 5, many provinces in Canada have more recently adopted variations of the MELD score to allocate organs, and across the country there exists significant variability in selection criteria for HCC patients and how exemptions are handled for tumour patients and other indications.

## 4. Discussion

In our validation study, the MELD score was significantly better at predicting waitlist mortality than the CanWAIT system. The AUROC for 3-month mortality approached 0.80 indicating that it is a good prognostic test for predicting short-term mortality on our LT waitlist. The AUROC curve for the MELD score was similar to other validation studies, in which AUROC curve ranged from 0.78 to 0.87 for the prediction of 3-month mortality [5–8]. In contrast, the AUROC curve for the CanWAIT algorithm was no better than chance alone at predicting waiting list mortality (95% CI included 0.5). The ability of the MELD score to predict longer-term mortality in Alberta was diminished, but the AUROC curve of 0.7 indicates that the MELD is still a useful model for predicting 1-year waitlist mortality. The inclusion or exclusion of ALF patients did not dramatically change the AUROC curves, and although not used for allocation in this patient population, there is evidence that the MELD score is a useful predictor of survival in patients with ALF [12].

Limitations of our study include its retrospective nature and the relatively small sample size of this single center experience. There were only small numbers of patients listed in the higher CanWAIT status categories. Another limitation was the inclusion of status 0 patients in the analysis. Although status 0 patients cannot receive a LT until they are activated, it was important to include these potential LT recipients to capture all wait time and all patients who died or were delisted for being too ill (15 out of 49 removals from the list were in status 0 patients).

Since MELD was adopted for LT allocation in the USA, many groups have examined if improvements can be made in the predictive capabilities of the model by refitting the coefficients of the existing variables or adding new variables to the model. Hyponatremia is an independent predictor of mortality in cirrhotics, and it has been suggested that addition of sodium to the model (MELD-Na) could potentially prevent 7% of waitlist deaths [13]. The MELD-Na has been adopted by the four adult LT programs in Ontario and Quebec. The MELD and MELD-Na have been refit to better predict mortality in patients awaiting LT [14]. Halifax has been using the refit MELD-Na for allocation since 2012. The predictive models for LT waitlist mortality have recently been reviewed by the Scientific Registry of Transplant Recipients (SRTR), and it appears that MELD-Na (with updated coefficients) is the best model for organ allocation (personal communication from Dr. Ray Kim). UNOS will be moving to adopt MELD-Na for LT allocation in the USA in the near future, and at the CLTF-9 meeting the Canadian LT Network voted to endorse MELD-Na (with the SRTR coefficients) as the universal allocation system for adult LT in Canada beginning on January 1, 2015. Therefore, the CanWAIT system now ceases to exist, although data on the location of the patient at the time of transplant (home, hospital, or ICU) will continue to be tracked.

In October 2005, Canadian LT hepatologists and surgeons met at the first CLTF in Montreal to discuss one fundamental question:* Should Canada adopt the MELD as a means of organ allocation?* Our validation study, which confirmed the ability of the MELD scoring system to predict short-term waiting list mortality in a cohort of Canadian patients, was presented at that meeting. As MELD was shown to be superior to the current CanWAIT system in predicting death while awaiting LT, there was universal support for adopting MELD for LT allocation in Canada. However, without an equivalent agency to UNOS in Canada, things have moved slowly, and as our 2014 survey indicates, MELD has been adopted in various forms across the country over the subsequent years.

Furthermore, there remains considerable variability in listing practices for HCC and how MELD exemptions are handled. BC and Atlantic Canada transplant HCC patients who are mainly within the Milan criteria (single HCC ≤ 5 cm or up to three HCC each ≤ 3 cm), although they will consider tumours within the UCSF criteria (single HCC < 6.5 cm or up to three HCC, none that are >4.5 cm, and cumulative tumour size <8 cm), on a case-by-case basis. In Alberta, since 2007, we have been transplanting HCC patients using a total tumour volume (TTV) of ≤115 cm^3^ *and* alpha-fetoprotein (AFP) ≤400 ng/mL as selection criteria [15, 16]. These criteria were subsequently adopted by the London program and then Ontario as a provincial policy in 2012, although patients can still be transplanted within UCSF criteria as well. In Quebec, they have a unique method of delivering MELD-Na exception points to some HCC patients, with this being assessed every three months [17]. How HCC patients are moved up the waitlist with exemption points is also not standardized in Canada (Table 5). However, after reviewing the prospective validation of TTV115 + AFP400 criteria at the CLTF-8 meeting (October 26, 2013, in Montreal) there was consensus to move forward with adopting this as a universal listing criterion for HCC in Canada.

**Figure 5:**
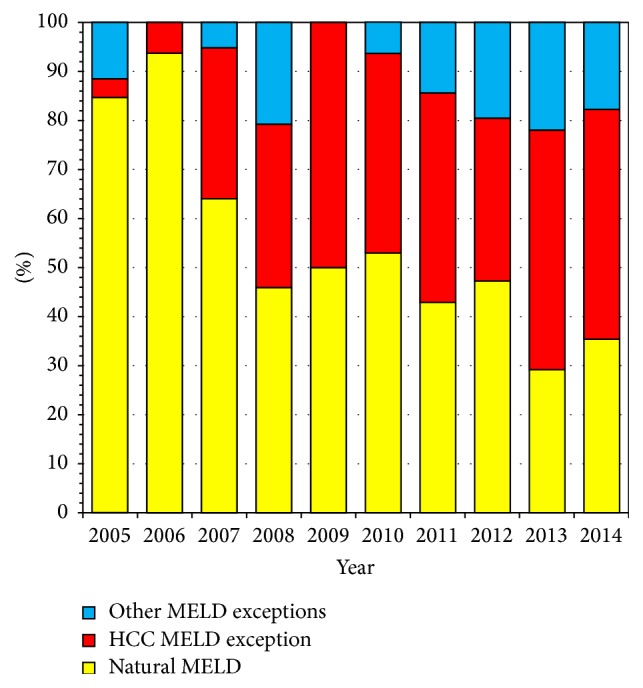
Percentage of adult LT in Alberta transplanted according to natural MELD versus exception points for HCC or other indications since adopting MELD based allocation.

A look at our adult patients in Alberta since we moved to MELD based allocation indicates that the majority of our patients are currently transplanted with MELD exception points, either for HCC or for other indications such as recurrent cholangitis, hepatopulmonary syndrome, or portopulmonary hypertension (Figure 5). Canadian Blood Services (CBS) is currently planning a LT Consensus Workshop in early 2016 to move forward the process of standardizing LT allocation policy, listing criteria, and how to handle MELD-Na exemptions in Canada. This will be extremely important if we wish to have universal and equitable access to LT in our country.

In conclusion, MELD is superior to the CanWAIT system for predicting waitlist mortality and has been validated in Canadian cohort of patients awaiting LT. Several modifications of MELD have subsequently been developed, and starting in January 2015, Canada has adopted MELD-Na (with SRTR coefficients) for LT allocation. There remains considerable heterogeneity in listing criteria and how MELD exceptions are handled in Canada and further consensus building workshops, along with a functional data management system, will be required to move this process forward.

## Figures and Tables

**Table 1:** Child Pugh (CP) classification.

Variable	1 point	2 points	3 points
Ascites	None	Easily controlled	Poorly controlled
Encephalopathy	None	Grade 1 or 2	Grade 3 or 4
Albumin (g/L)	<35	28–35	<28
Bilirubin (*μ*mol/L)	<34	34–51	>51
INR	<1.7	1.7–2.3	>2.3

Abbreviation: INR, international normalized ration of prothrombin time.

**Table 2:** CanWAIT allocation system.

CanWAIT	Definition
4F	ALF in ICU on ventilator
4	Chronic liver disease in ICU on ventilator
3F	ALF in ICU not requiring mechanical ventilation

3	Chronic liver disease in ICU for Grade 3 or 4 encephalopathy or renal dysfunction but not requiring ventilation
2	Chronic liver disease in hospital
1T	Chronic liver disease at home with HCC
1	Chronic liver disease at home
0	On hold for liver transplantation

*Note*: Organs were shared nationally for urgent status (3F, 4, 4F) until May 2010 after which national sharing was restricted to patients with acute liver failure only (3F, 4F).

Abbreviations: ALF, acute liver failure; ICU, intensive care unit; HCC, hepatocellular carcinoma.

**(a):** 

MELD	*N*	% Total	Deaths	% Mortality
<10	92	28.8%	7	7.6%
10–19	160	50%	22	13.8%
20–29	46	14.4%	11	23.9%
30–39	15	4.7%	7	46.7%
≥40	7	2.2%	2	28.6%

**(b):** 

CP class	*N*	% Total	Deaths	% Mortality
A (5-6)	32	10%	3	9.4%
B (7–9)	145	45.3%	10	6.9%
C (10–15)	143	44.7%	36	25.2%

**(c):** 

CanWAIT status	*N*	% Total	Deaths	% Mortality
0	105	32.8%	15	14.3%
1	146	45.6%	20	13.7%
1T	9	2.8%	2	22.2%
2	43	13.4%	7	16.3%
3	4	1.3%	1	25%
3F	1	0.3%	0	0%
4	4	1.3%	2	50%
4F	8	2.5%	2	25%

**Table 4:** Cox proportional hazards models for 1-year wait list mortality for different MELD strata.

MELD	*N*	HR	(95% CI)	*p* value
<10	92	—	—	—
10–19	160	1.74	(0.74, 4.12)	0.207
20–29	46	8.48	(3.17, 22.64)	<0.0005
30–39	15	82.02	(24.28, 277.08)	<0.0005
≥40	7	54.88	(10.35, 290.95)	<0.0005

**Table 5:** Survey of MELD use in Canada (October 2014).

	BC	AB	ON	PQ	ATL
System	MELD and CanWAIT	MELD 20^*∗*^ and CanWAIT	MELD → MELD-Na	MELD-Na	MELD → Refit MELD-Na

Adopted	2006	JUL 2004	2006 → NOV 2012	JUL 2008	2006 → 2012

Criteria for HCC	Milan (UCSF)	TTV115 + AFP400	TTV115 + AFP400 or UCSF	Milan	Milan (UCSF)

HCC Exemptions	MELD 15 + 10% q3m	MELD 22 + 2 pts q3m	MELD-Na 22 + 3 pts q3m	PQ-HCC-MELD q3m	Refit MELD-Na 22

Other Exemptions	None	HPS, PPHT, Cholangitis, and others	HPS, FAP, HB, 1°HO, CF, metabolic, CCA, Failed LDLT or DCD	Cholangitis, HE, HPS, HEHE, and others	Cholangitis, PCLKD, and others

Abbreviations: BC, British Columbia; AB, Alberta; ON, Ontario; PQ, Quebec; ATL, Atlantic Canada; MELD, model of end-stage liver disease; MELD-Na, MELD-sodium; UCSF, University of California San Francisco; TTV115, total tumour volume ≤ 115 cm^3^; AFP400, alpha-fetoprotein ≤ 400 ng/mL; q3m, every three months; HPS, hepatopulmonary syndrome; PPHT, porto-pulmonary hypertension; FAP, familial amyloidosis polyneuropathy; 1°HO, primary hyperoxyluria; CF, cystic fibrosis; CCA, cholangiocarcinoma; LDLT, live donor liver transplantation; HE, hepatic encephalopathy; HEHE, hepatic epitheliod hemangioendothelioma; PCLKD, polycystic liver and kidney disease.

^*∗*^MELD 20 policy (July 2004–December 2014) = Patients waiting at home (status 1) with a MELD ≥ 20 were given priority; however, hospitalized patients (status 2) with a lower MELD score would still receive an organ first.
